# Association between obstructive sleep apnea and resistant hypertension: systematic review and meta-analysis

**DOI:** 10.3389/fmed.2023.1200952

**Published:** 2023-06-02

**Authors:** Abass Mahamoud Ahmed, Salman Mohamud Nur, Yuan Xiaochen

**Affiliations:** ^1^Department of Cardiology, Affiliated Hospital of Yangzhou University, Yangzhou University, Yangzhou, Jiangsu, China; ^2^Medical College, Yangzhou University, Yangzhou, Jiangsu, China; ^3^Department of Neurology, Affiliated Hospital of Yangzhou University, Yangzhou, Jiangsu, China

**Keywords:** obstructive sleep apnoea (OSA), resistant hypertension, apnea-hypoxia index (AHI), risk factors, hypoxia

## Abstract

**Introduction:**

Obstructive sleep apnea syndrome (OSAS) is a chronic disorder characterized by recurring episode obstruction and collapse of upper airways during sleep, leading to hypoxia and sleep disruption. OSAS is commonly associated with an increased prevalence of hypertension. The underlying mechanism in OSA with hypertension is related to intermittent hypoxia. This hypoxia induces endothelial dysfunction, overactivity of sympathetic effects, oxidative stress, and systemic inflammation. Hypoxemia triggers the sympathetic process's overactivity, leading to the development of resistant hypertension in OSA. Thus, we hypothesize to evaluate the association between resistant hypertension and OSA.

**Methods:**

The PubMed, ClinicalTrails.gov, CINAHL, Google Scholar, Cochrane Library, and Science Direct databases were searched from 2000 to January 2022 for studies demonstrating the association between resistant hypertension and OSA. The eligible articles underwent quality appraisal, meta-analysis, and heterogeneity assessment.

**Results:**

This study comprises seven studies, including 2,541 patients ranged from 20 to 70 years. The pooled analysis of six studies demonstrated that OSAS patients with a history of increased age, gender, obesity, and smoking status are at an increased risk for resistant hypertension (OR: 4.16 [3.07, 5.64], *I*^2^:0%) than the non-OSAS patients. Similarly, the pooled effect demonstrated that patients with OSAS were at an increased risk of resistant hypertension (OR: 3.34 [2.44, 4.58]; *I*^2^:0%) than the non-OSAS patients when all associated risk factors were adjusted using multivariate analysis.

**Conclusion:**

This study concludes that OSAS patients with or without related risk factors demonstrated increased risk for resistant hypertension.

## Introduction

Obstructive sleep apnea (OSA) is a highly prevalent and chronic disorder characterized by the persistent partial or complete collapse of the upper airway during sleep, resulting in hypoxia and sleep disruption (1). In its seventh report, The Joint National Committee on Prevention, Detection, Adults patients, Evaluation, and Treatment of hypertension reported OSA as a significant cause of resistant hypertension (2).

The prevalence of OSA ranges from 2 to 4% in middle-aged men and 2% in middle-aged females. The OSA prevalence continues with increasing age and obesity (3, 4). Other associated risk factors for OSA include large uvula, cardiovascular disease, retrognathia, macroglossia, obstructive nasal airway, and larger neck size (>40 cm in females and >42 cm in males) (4).

The risk ratio of females to males in the general population and population with other comorbidities ranges from 1:3 to 5:11 and 1:8 to 1:10 (5). Women suffer from OSA at an older age with few apneic events and are less severe than men. Young adults (< 50 years) have a strong association for OSA with hypertension than older adults (>50 years) (6).

A randomized clinical trial (RCT), the Heart Biomarker Evaluation in Apnea Treatment (HeartBEAT) study, conveyed that moderate OSA was associated with a low prevalence of difficult-to-treat hypertension than untreated severe OSA (7). Resistant hypertension usually occurs in 12–15% of treated hypertensive patients (8). Hypertensive patients with OSA have a significant incidence of attenuated nocturnal BP fall. The Wisconsin Sleep Cohort Study demonstrated a significant association between OSA and nocturnal BP non-dipping behaviors with 7.2 years of an average follow-up (9).

Hypertension is a major cause of cardiovascular disease, stroke disability, and mortality globally. Resistant hypertension is characterized as above-desired elevated blood pressure in patients that persist despite the concurrent use of at least three or more antihypertensive drugs, including diuretics, calcium channel blocker, and angiotensin receptor blockers or angiotensin-converting enzyme inhibitors administered at the maximum tolerated dose and frequency. Also, patients with non-adherence to antihypertensive therapy, pseudo resistance, or white-coat effects are always excluded from the resistant hypertension category (10, 11). Similarly, resistant hypertension also includes patients with controlled hypertension who consume four classes of prescribed antihypertensive drugs (11).

The “National Health and Nutrition Examination Survey (NHANES) 2003–2008” reported that 12.8% of US hypertensive patients receiving antihypertensive medication suffered from resistant hypertension (12). The prevalence of patients treated for resistant hypertension has increased from 15.9% in 1998–2004 to 26% in 2019 (13, 14). Patients with resistant hypertension have a significantly higher risk for cardiovascular complications and organ failure than non-resistant hypertensive patients (15). Thus, managing the patient's prognosis with resistant hypertension becomes essential. The associated risk factors for the development of resistant hypertension include advanced age, female gender, diabetes mellitus, obesity, alcohol, dietary sodium, physical inactivity, chronic kidney disease, sleep disorder, pseudopheochromocytoma, dietary non-compliance, NSAID use, and sleep apnea (14).

Several studies have reported obstructive sleep apnea (OSA) as a strong risk factor for the development of resistant hypertension (10, 16), with 83% of OSA patients presenting to the clinic for resistant hypertension (17). Similarly, Calhoun et al. (18) revealed that OSA risk based on Berlin questionnaires was higher among 63% of patients presenting to clinics for resistant hypertension. Goncalves et al. (19) revealed that OSA patients have nearly 5 times more risk for resistant hypertension than non-OSA patients. Lavie et al. (20) in a prospective study reported that the severity and prevalence of hypertension increase with an increase in an apnea–hypopnea index (AHI).

Apnea–hypopnea index (AHI) measures the frequency of apnea and hypopnea per hour. The American Academy of Sleep Medicine defines OSA diagnosis criteria as >15 AHI without OSA symptoms or >5 AHI with OSA symptoms (1). Several observational studies have demonstrated that OSA prevalence is about 80% in resistant hypertensive individuals and more than 30% in hypertension patients (21, 22). Some literature failed to demonstrate the association between hypertension and OSA despite the obvious association existing between both (23). More than eight studies have reported no association between hypertension and OSA, provoking incredulity of the OSA effect on hypertension risk (24, 25).

We hypothesized that OSA patients are strongly associated with resistant hypertension than non-OSA patients.

## Methodology

### Design

This study evaluates the relationship between OSAS and resistant hypertension following Preferred Reporting Items for Systematic Review and Meta-analysis (PRISMA) (26).

### Search strategy and database

Two authors explored PubMed, ClinicalTrails.gov, CINAHL, Google Scholar, Cochrane Library, and Science Direct databases from 2000 to January 2022. We performed an electronic search across these databases to discover the potential articles using keywords: “sleep disordered breathing,” “obstructive sleep apnea syndrome,” “OSAS,” “obstructive sleep apnea,” “OSA,” “sleep apnea,” “Hypertension,” “HTN,” “resistant hypertension,” “Uncontrolled hypertension,” and “high blood pressure.” We used Boolean operators and relevant keywords to find their intersection. Also, search keywords were matched based on a different database. Manually, we checked the reference of eligible studies, relevant abstracts, and narrative reviews.

### Study criteria

Only articles that satisfy the following study criteria were considered: (1) randomized control trials or observational studies demonstrating the association between OSAS and resistant hypertension; (2) patients with OSA or resistant hypertension; (3) adults patients; (4) both gender; (5) articles demonstrating AHI. The search for articles was limited to humans and the English language.

The articles were excluded if the article satisfied the following criterion (1) review papers; (2) no full text available; (3) editorial letter/commentaries; (4) non-research letter; (5) animal studies; (6) case reports or case series; (7) conference abstracts; (8) papers without a quantitative measure of AHI; (8) high-sodium diet food; (9) white coat effect; (10) non-compliant to antihypertensive therapy.

### Data synthesis, outcome assessment, and statistical analysis

Two authors independently reviewed each recognized article to exclude the articles that failed to satisfy the research criteria. An independent third-party reviewer resolved any discrepancies. We did a full-text analysis if the potentially relevant article's abstract could not provide precise conclusions. The inclusion and exclusion criteria were utilized to identify the eligible studies and obtain the full-text articles. We selected eligible studies irrespective of publication and authors of papers. We extracted data after a full-text assessment. We designed a standardized data collection form using Microsoft Word. Two writers independently collected article details, including country, study design, study period, total sample size, age, BMI, and standard BP measuring instrument.

We measured the primary outcome as an association between OSA and resistant hypertension using AHI event or number of AHI episodes per hour.

We used comprehensive meta-analysis (CMA) software version 3 to estimate the overall effect of primary outcomes and create a forest plot to present the outcomes (27). The pooled effect was considered statistically significant at a *p*-value < 0.05. This study assesses the standard difference (MD), and odds ratio (OR) derived from the mean, standard deviation, confidence interval, and sample size. *I*^2^ test assessed the heterogeneity level. The degree of heterogeneity level was categorized into minimal (*I*^2^ < 30%), moderate (*I*^2^: 30–60%), and substantial (*I*^2^ < 100%). Fixed-effects model was used in estimating overall effects for low or minimal heterogeneity, whereas random-effects model was used in estimating overall effects for moderate and substantial heterogeneity.

### Quality appraisal

**Figure 1:**
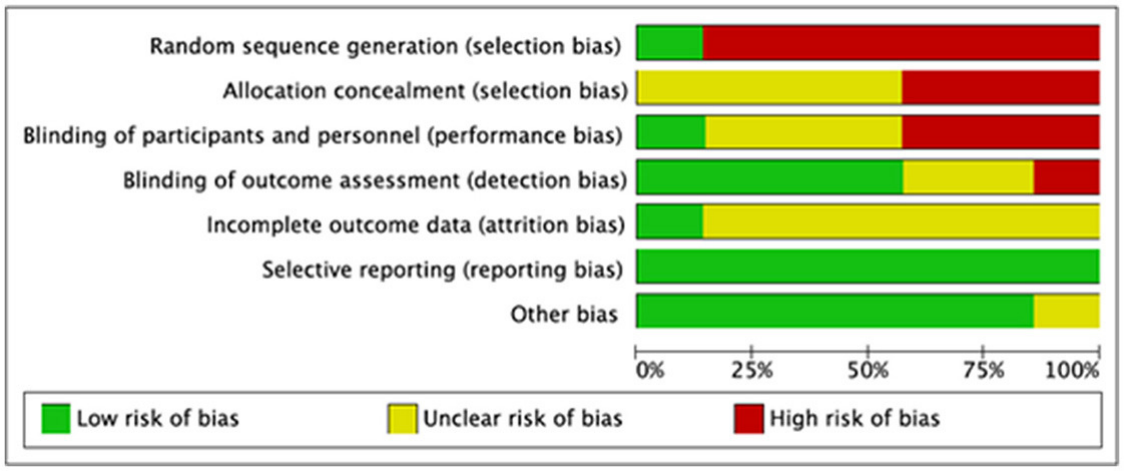
Risk of bias graph.

**Figure 2:**
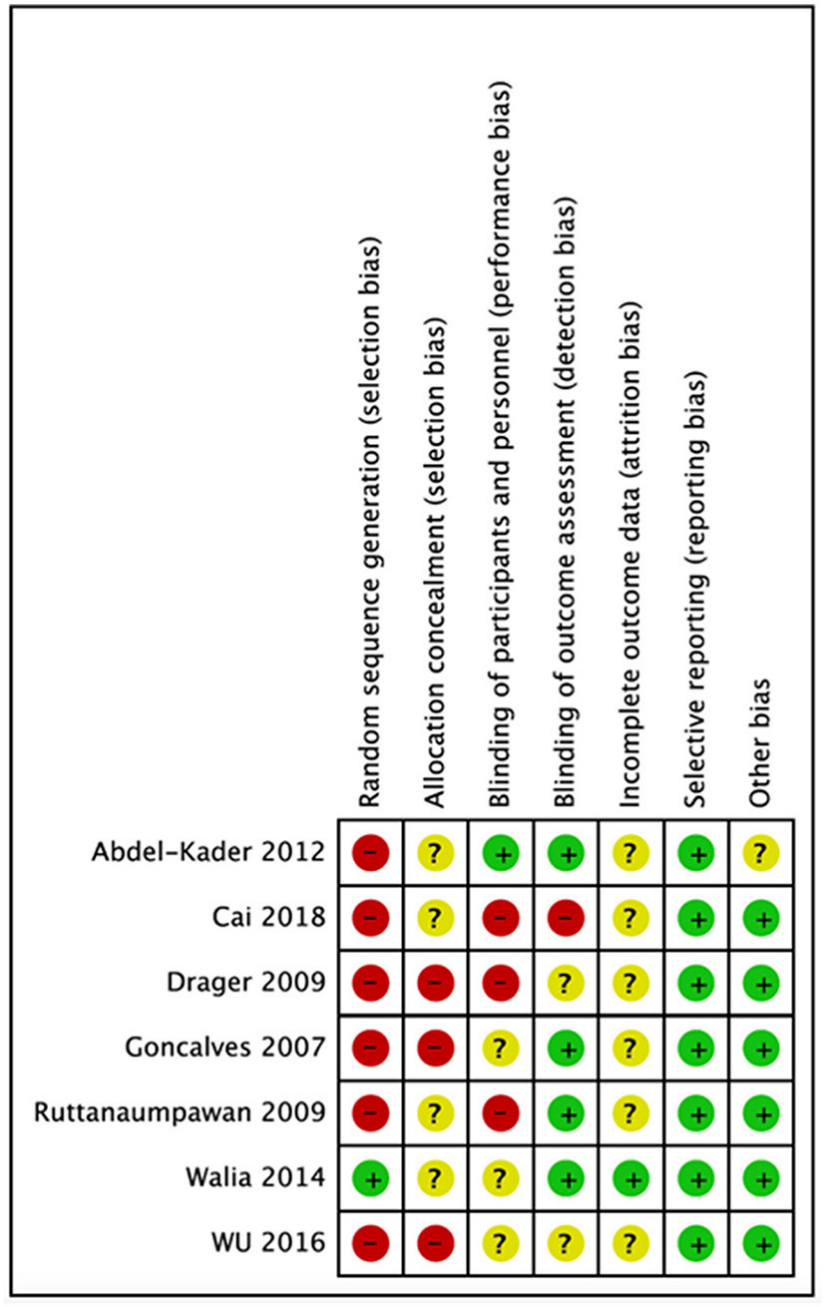
Risk of bias summary for each study.

We evaluated the quality of seven studies—Goncalves et al. (19), Abdel-Kader et al. (30), Cai et al. (31), Ruttanaumpawan et al. (32), Walia et al. (7), Wu et al. (33), and Drager et al. (34)—using the Cochrane risk-of-bias tool. A popular method for determining the risk of bias in studies is the Cochrane risk of bias tool. It assesses the study's quality using a variety of criteria, including selection bias, performance bias, detection bias, attrition bias, reporting bias, and other biases that can affect the findings. The tool aids in the identification of possible biases and restrictions in the planning and execution of studies as well as the evaluation of the general validity and dependability of the research results. We were able to evaluate the seven studies' quality, find any possible biases and limitations, and calculate the total risk of bias for each research by utilizing this technique to perform a quality evaluation. Making conclusions concerning the applicability and usefulness of the study's results requires knowledge of this information. Following that, each study's risk of bias graph and risk of bias summary are shown in Figures 1, 2.

## Results

### Characteristics of study and literature search

**Figure 3:**
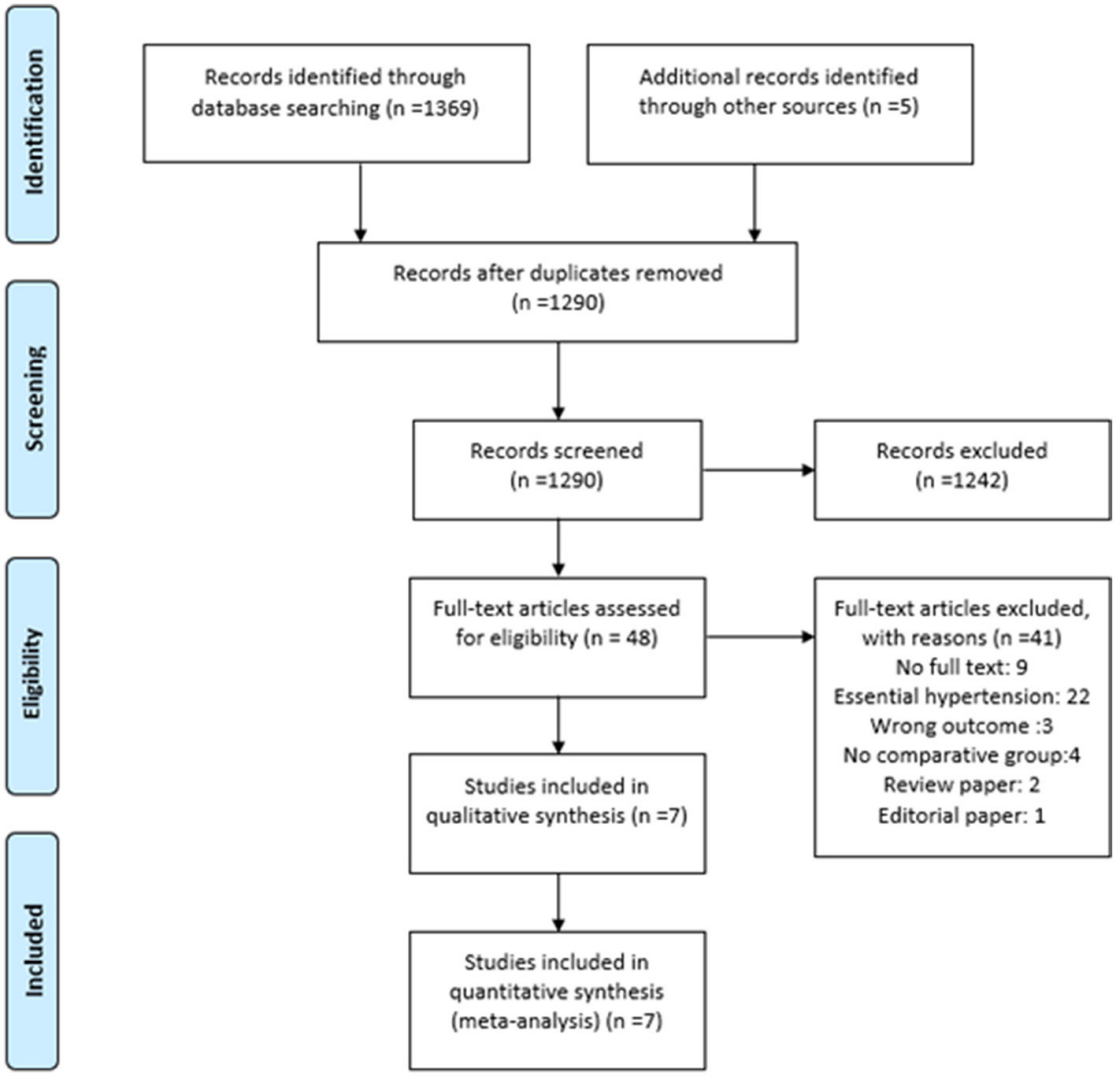
PRISMA flow diagram to demonstrate the article search and the inclusion process.

We identified 1,374 articles across all databases using search keywords and Boolean operators. Duplicate records were removed with endnote v20 (28) using similar author names, publication years, journal names, and article titles. Two reviewers independently removed 1,242 articles, leaving 48 articles for full-text analysis. Only seven articles fulfilled our study criteria. The flowchart for the literature search process is presented as PRISMA flow (29) in Figure 3.

In all, five studies reported a cohort comparison, one randomized control trial, and one case–control study among seven eligible studies. These seven studies assessed the association between OSA and resistant hypertension (7, 19, 30–34), including 2,541 patients. The characteristics of all eligible studies are summarized in Table 1.

**Table 1:** Study characteristics of included studies.

**Study ID**	**Authors**	**Country**	**Study designs**	**Study period**	**Case**	**Sample size (case/control)**	**Age (case/control) in years**	**BMI (case/control)**	**Adjusted variable**
Doi: 10.1378/chest.07-117	Goncalves et al. (19)	Brazil	Case control	March 2004–June 2006	Hospital-based R-HTN	63/63	59 ± 7/59 ± 7	30 ± 3/29 ± 4	Age, gender, BMI, hypertension duration
Doi: 10.1097/HJH.0b013e328351d08a	Abdel-Kader et al. (30)	NR	Cohort study	March 2004- December 2008	Community-based R-HTN	224	60 ± 7.2	29.5 ± 5.0	Age, gender, Race, BMI
Doi: 10.1016/j.ijcard.2017.10.089	Cai et al. (31)	China	Cohort study	February 2015–september 2016	Hospital-based R-HTN	1157	56.6 ± 11.7	24.4 ± 4.7	Age, Gender, Neck Girth, BMI,
Doi: 10.1097/HJH.0b013e32832af679	Ruttanaumpawan et al. (32)	Canada	Cohort study	NR	Hospital-based R-HTN	42/22	56.5 ± 1.6/60.1 ± 1.8	33.7 ± 0.9/32.9 ± 1.0	Age, sex and BMI
Doi: 10.5664/jcsm.3946	Walia et al. (7)	US	RCT	February 2010–august 2012	Hospital based OSA	28/175	65.2 ± 6.5/62.5 ± 7.4	36.3 ± 5.3/33.9 ± 5.4	Age, sex, race, body mass index, smoking status
Doi: 10.3109/10641963.2016.1151525	Wu et al. (33)	South China	Cohort study	April 2008–August 2012	Hospital based OSA	216/452	54.3 ± 3.0	28.07 ± 3.72	Gender, age, BMI, and duration Of hypertension
Doi: 10.1016/j.amjcard.2009.12.017	Drager et al. (34)	Brazil	Cohort study	January 2009–July 2009	Hospital based OSA	55/44	51 ± 10/40 ± 10	30.95 ± 1.80/26.95 ± 1.69	Age, gender, BMI

ABPM, ambulatory BP monitoring.

### Major findings of eligible studies

Goncalves et al. (19) assessed the association of OSAS with resistant hypertension in a case–control study conducted at Hospital de Clínicas de Porto Alegre, Brazil. This study included 126 patients allocated equally into the case and control arms. Participants in the case arm included resistant hypertension, while the controlled arm included controlled resistant hypertension. The study sample size of 62 patients in each group was calculated based on the expected frequency of OSAS of 70% among resistant hypertension patients and 40% among controlled hypertension patients with an a-error of 5% and 90% power. The primary outcome was the frequency of OSAS in the case and control arm. The OSAS prevalence with AHI ≥10 per hour in the case and control groups was 71% and 38%, respectively. Similarly, multiple logistic regression analysis suggested that OSAS was independently associated with resistant hypertension (OR: 4.8 [2.0–11.7]). This study also provides evidence as OSAS is an independent risk factor for resistant hypertension (19).

Abdel-Kader et al. (30) conducted a single-center, prospective community-based cohort study to assess the relationship between resistant hypertension and OSAS in renal and non-renal disease patients. The systolic blood pressure was significantly higher among renal patients than non-renal patients. At the same time, diastolic blood pressure did not differ significantly between the two groups. The adjusted (for age, gender, BMI, and race) and odds unadjusted ratio demonstrated a significant association between resistant hypertension and obstructive sleep apnea among end-stage renal disease. Abdel-Kader et al. (30) concluded that renal disease patients had an increased risk for OSAS and resistant hypertension. Moreover, OSAS patients with comorbidities like end-stage renal disease are 7.1 times more likely to develop resistant hypertension (OR: 7.1 [2.2, 23.2]). Similarly, obstructive sleep apnea demonstrated an increased trend toward the odds for resistant hypertension in non-chronic kidney disease (30). Also, Drager et al. (34) assessed 99 patients for the association between OSA and hypertension. In total, 55 patients were included in the OSA arm and the remaining 44 in the non-OSA arm. Drager et al. concluded that OSA patients have a 7.7 higher risk for resistant hypertension (OR: 7.74 [2.24, 24.64]) (34).

Cai et al. (31) performed a cross-sectional study to access the combined effect of resistant hypertension and OSAS on chronic heart failure in 1,157 patients. This study reported that 33.1% of participants had OSAS. The prevalence of resistant hypertension in OSAS patients was 18.3%. The resistant hypertension patients had a mean blood pressure of 151 ± 20/87±14 mmHg and received 3.5±0.6 average antihypertensive drugs. The prevalence of resistant hypertension in OSAS patients with AHI> 15 events/hours (18.3%) was significantly higher than in participants with AHI < 5 events/hours (7.6%) and AHI 6–14 events/hours (12.7%). Therefore, the prevalence of resistant hypertension was progressively increasing with the severity of OSAS. Moreover, multiple regression analysis demonstrated that an increase in the severity of OSAS independently increased the risk of resistant hypertension (OR: 1.05 [1.02, 1.08]) after adjusting for covariates including gender, age, obesity, serum uric acid, diabetes mellitus, neck girth, average SaO_2_ level, and heart failure (31). The same study also revealed that the prevalence of resistant hypertension increased by 15.9% (95% CI 10.0%−21.8%) with every increase of AHI by five events/hour and decreased SaO_2_ by 1.71% (31).

Similarly, Ruttanaumpawan et al. compared the prevalence of OSA and REM sleep time between controlled hypertension and resistant hypertension in 42 patients. Patients with resistant hypertension have significantly demonstrated a higher prevalence of OSA (81% *vs*. 55%, *p*: 0.03) than patients with control hypertension. Moreover, multi-regression concluded that OSA independently increased the risk of resistant hypertension (OR: 3.9 [1.19, 13.38]) (32).

Similarly, Walia et al. (7) performed a randomized control trial with 284 participants to assess the association of resistant hypertension. This study concluded that the severity of OSAS is directly associated with resistant hypertension. Severe OSAS patients significantly demonstrated a higher prevalence of resistant hypertension (58.3% vs. 28.6%, *p*: 0.01) than the moderate OSAS. The multivariate analysis concluded that severe OSAS patients have four times more risk of developing resistant hypertension (OR:4.1[1.7, 10.2]) (7).

Wu et al. (33) assessed the association between OSAS and resistant hypertension in 668 snorer patients at the People's Hospital of Guangxi Zhuang Autonomous Region, China. Apnea (OR: 0.97 [0.954–0.99], *p*: 0.015] and the presence of OSAS (OR: 1.82 [1.04–3.2], *p*: 0.03) significantly demonstrated as an independent risk factor for resistant hypertension (33).

### Results of meta-analysis

**Figure 4:**
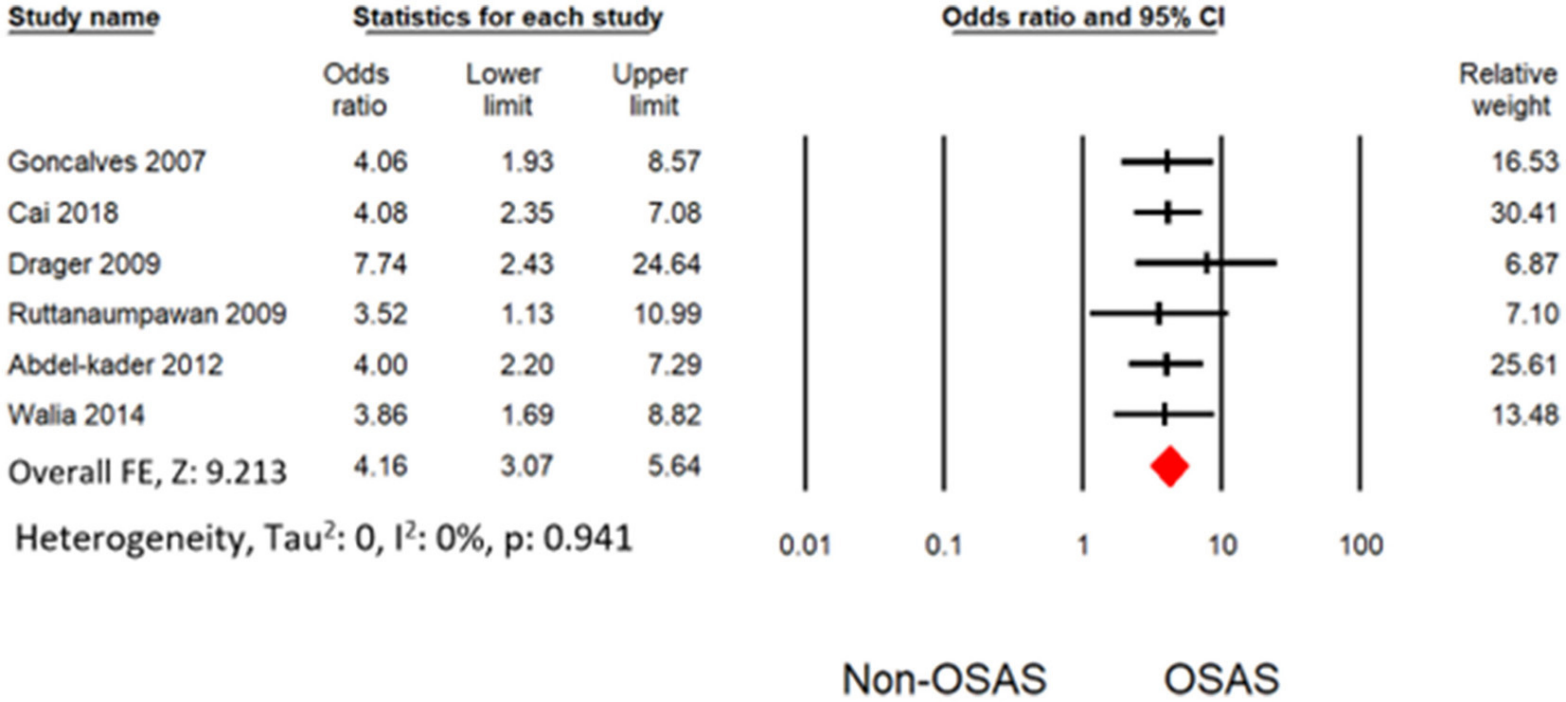
Forest plot illustrating an association between obstructive sleep apnea and resistant hypertension (results from univariate analysis).

**Figure 5:**
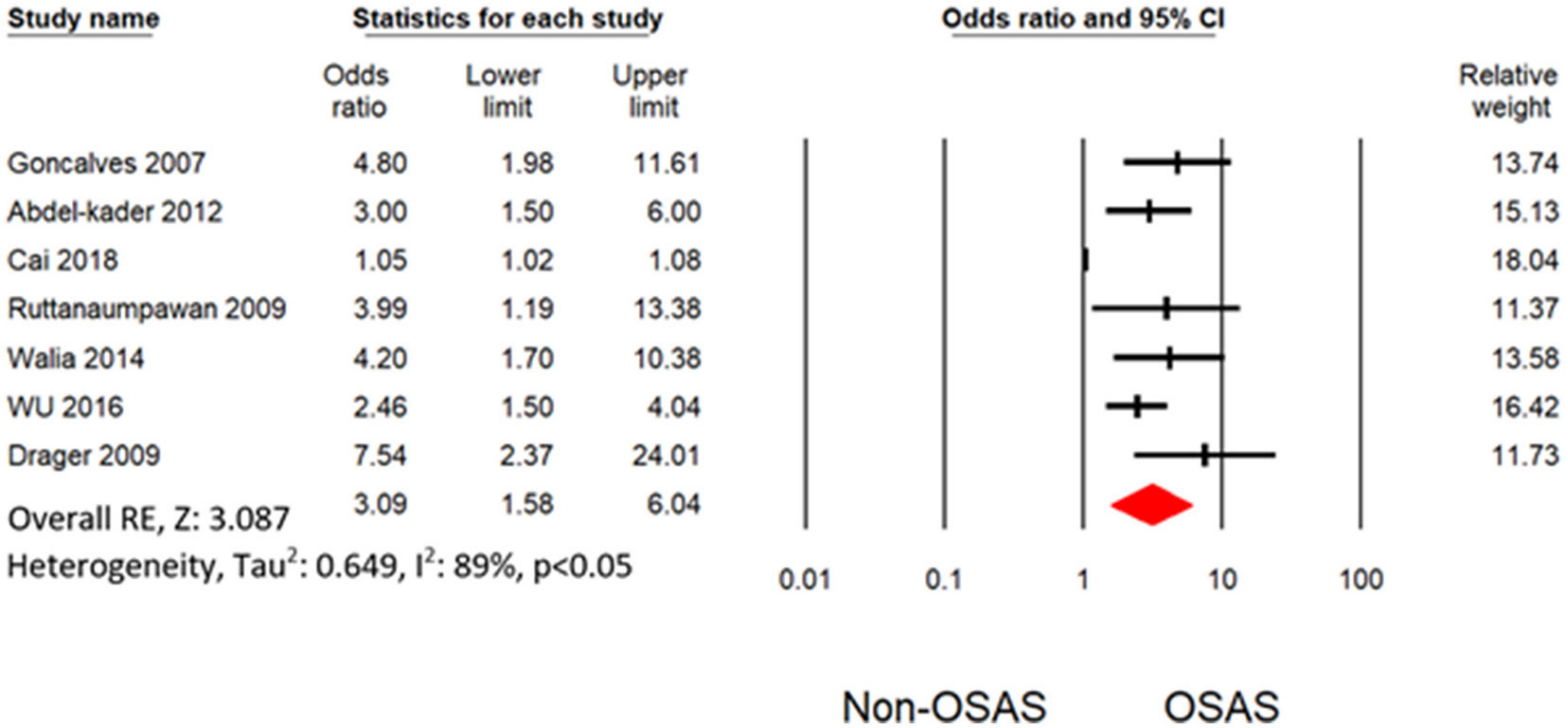
Forest plot illustrating an association between obstructive sleep apnea and resistant hypertension (results from multivariate analysis).

**Figure 6:**
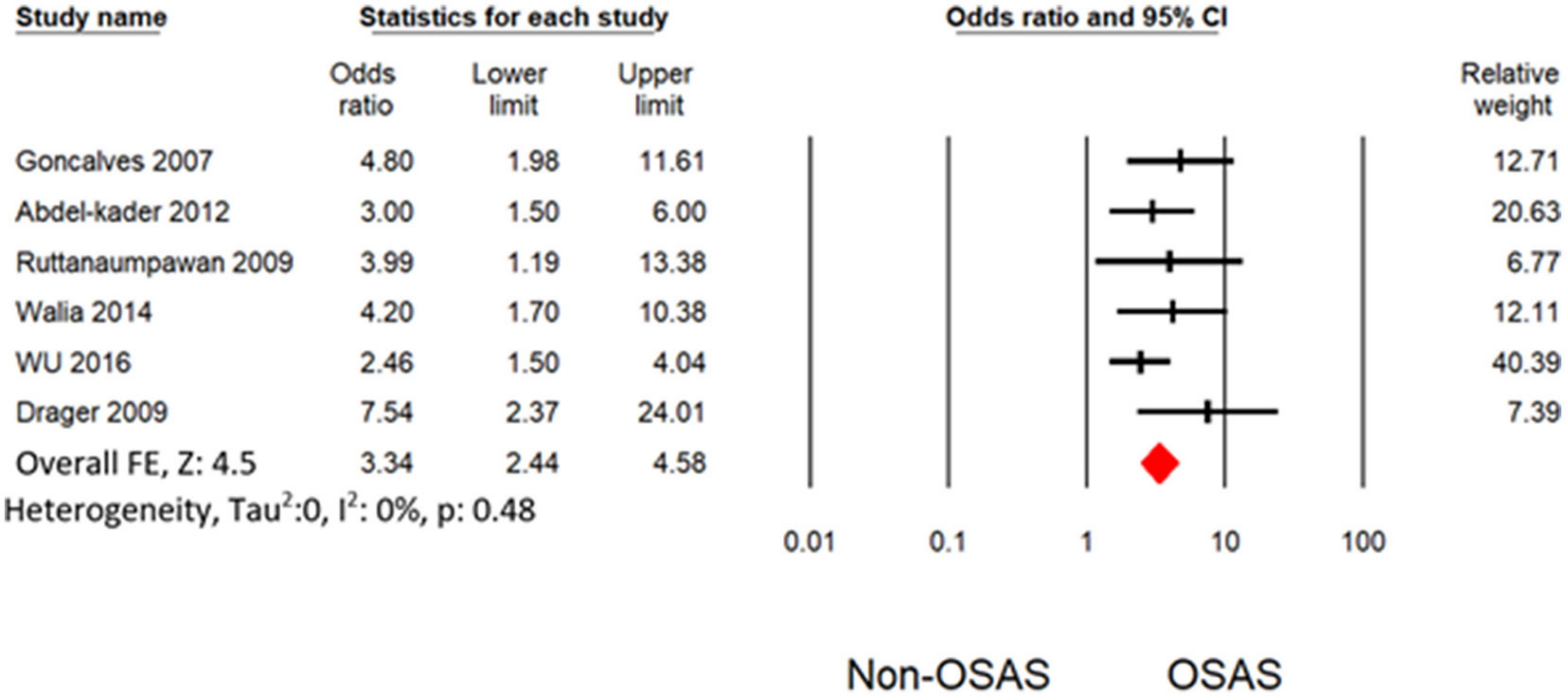
Forest plot illustrating an association between obstructive sleep apnea and resistant hypertension (results from multivariate analysis) by omitting Cai et al. (31).

Six studies measured the univariate association between resistant hypertension and OSAS (7, 19, 30–32, 34). The overall effects demonstrated that OSAS patients are four times more likely to develop resistant hypertension (OR: 4.16 [3.07, 5.64]) than non-OSAS patients, as in Figure 4. Similarly, the overall effect of multivariate analysis of the outcome of all eligible studies demonstrated that OSAS significantly increases the risk for resistant hypertension (OR: 3.09 [1.58, 6.04]) as in Figure 5 with substantial heterogeneity. Thus, we conducted sensitivity analysis by removing one article demonstrating higher heterogeneity across the group (31). The overall effect of multivariate analysis across each study demonstrated that OSAS significantly increases the risk of resistant hypertension (OR: 3.34 [2.44, 4.58]) with minimal heterogeneity (Figure 6).

## Publication bias

**Figure 7:**
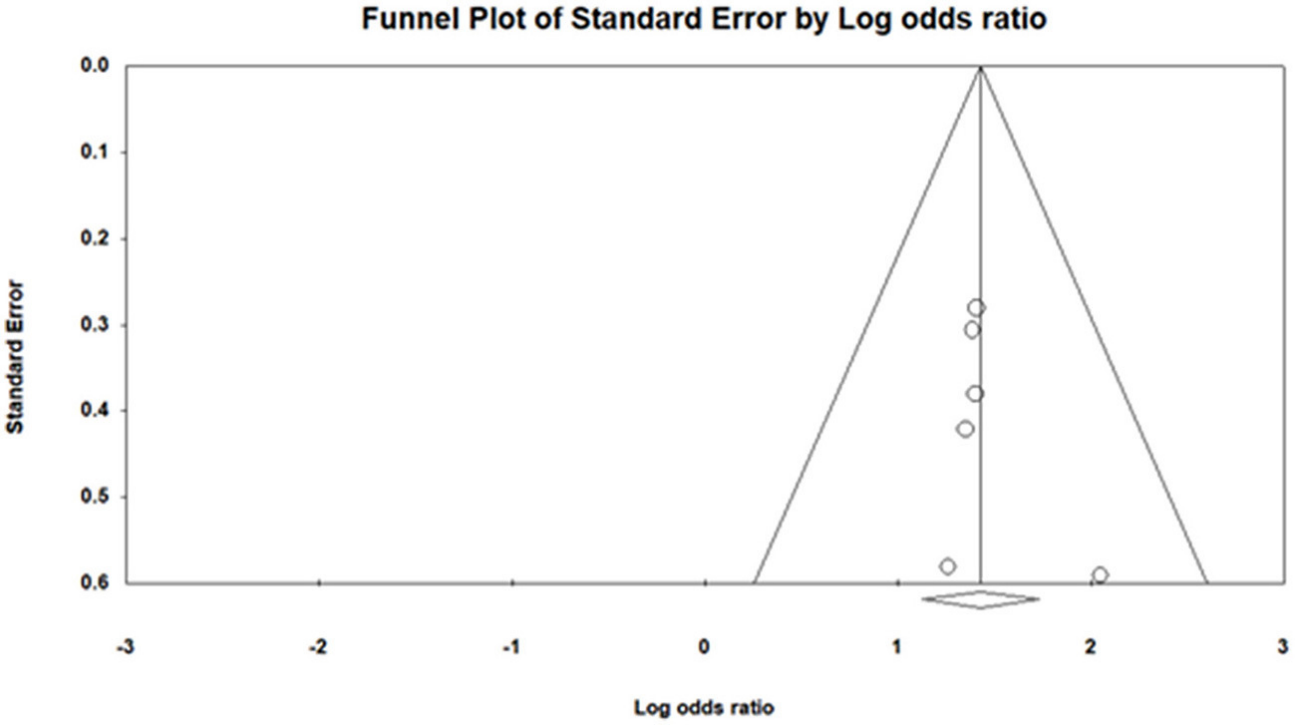
Publication bias.

We applied funnel plot analysis to detect the presence of publication bias. The funnel plot (Figure 7) of log odds ratio vs. standard error demonstrated the presence of the publication bias. Similarly, Egger's test also proved the existence of publication bias (intercept: 0.73 ±0.76, [−1.40, 2.86]), *t*-value: 0.95) (35).

## Discussion

**Figure 8:**
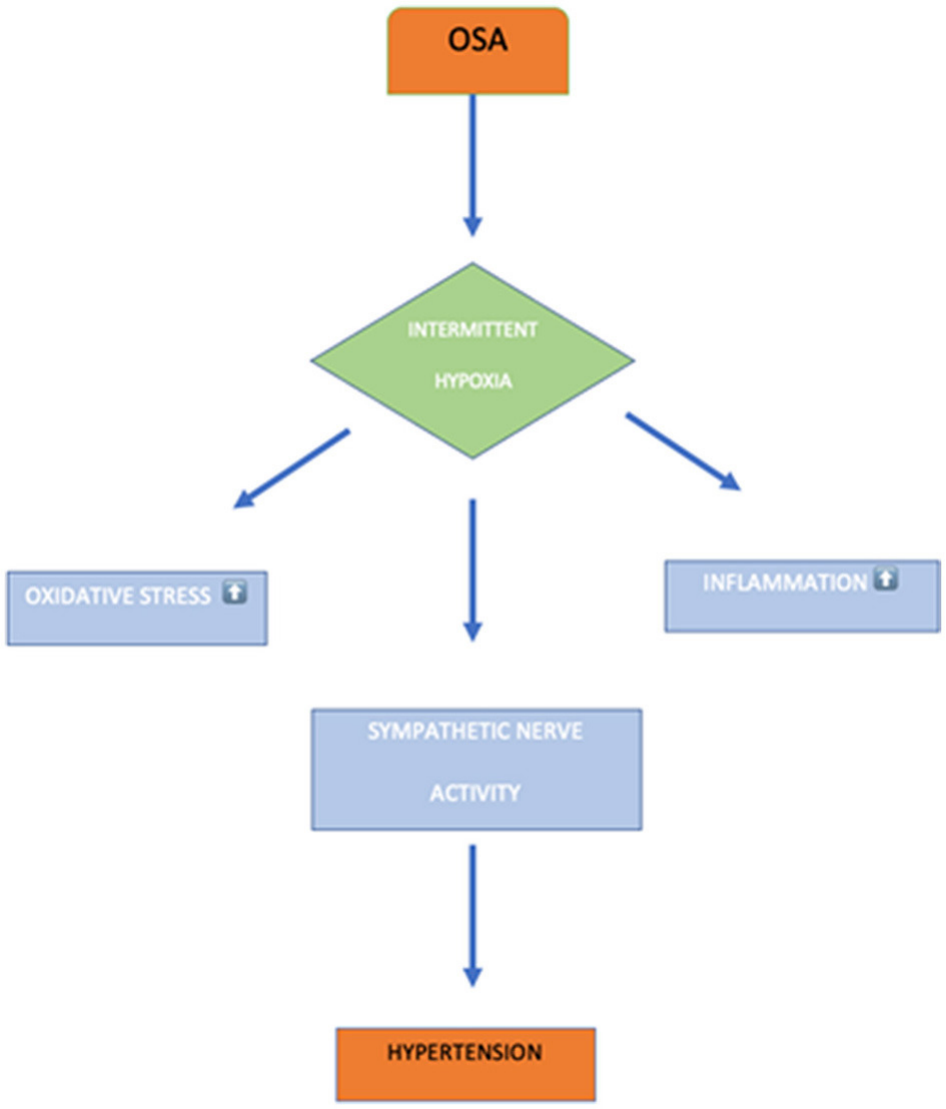
Schematic diagram showing how OSA causes hypertension.

This systematic review and meta-analysis were performed to evaluate the association between resistant hypertension and OSA, considering AHI >10 events per hour. This review finding suggests that OSAS is significantly associated with resistant hypertension. In addition, resistant hypertension was associated with the severity of OSAS. Patients with severe OSA symptoms had a higher risk for resistant hypertension (7). We performed the first meta-analysis that demonstrates an independent association between OSAS and resistant hypertension. The potential mechanism for OSAS association with resistant hypertension is yet to be understood. However, numerous potential theories may aid us in understanding the relationship between resistant hypertension and OSAS. OSA causes vascular endothelium dysfunction and oxidative stress that provoke intermittent hypoxia (36). In the meantime, hypertension is caused by excessive sympathetic vasoconstrictor effects and reduced bioavailability of nitric oxide (37, 38). Moreover, increasing AHI episodes per hour further stimulates the sympathetic system that affects the chemoreceptors to cause resistant hypertension. Thus, the combined effects of reduced nitric oxide and excessive sympathetic vasoconstrictors effects cause hypertension as shown in Figure 8 (38). The 2018 study by Haifeng Hou et al. (39) investigated the association between OSA and resistant hypertension and found that OSA participants had an extra 1.842-fold risk for resistant hypertension prevalence compared with non-OSA participants. This finding is consistent with our systematic review and meta-analysis, which is in favor of the same finding that OSA participants have an increased risk for resistant hypertension. Our study demonstrates that the majority of resistant hypertension in OSA is 4.1 times more than in non-OSA patients. Hou's and our research both discovered a link between OSA and hypertension, although the effect sizes were different.

According to Haifeng Hou's meta-analysis, OSA patients had a greater chance of developing hypertension than non-OSA patients (OR: 2.15 [1.80, 2.57]). On the other hand, compared with non-OSA patients, OSA patients had a 4-fold increased risk of resistant hypertension (OR: 4.16 [3.07, 5.64]). Our study's impact size is larger and more substantial, pointing to a more direct link between OSA and resistant hypertension. OSA substantially raises the risk of resistant hypertension according to our study's multivariate analysis (OR: 3.34 [2.44, 4.58]).

According to the schematic diagram, we elaborated on various reasons which cause OSA subsequently leading to hypertension. However, previous studies have further evaluated the BMI and obesity, which lead to OSA and then to systematic hypertension. For example, a study conducted by Carratù et al. (40) suggested in their results that in comparison with lean and overweight subjects, obese subjects displayed higher rates of systemic hypertension, type 2 diabetes, higher serum total cholesterol concentrations, and lower HDL cholesterol concentrations.

To further stratify this point, another study by Carratù et al. (41) conducted in 2016 presented the same verdict and findings which showed that OSA is linked to the weight and BMI of patients, and according to the sample of 97 individuals in that study, the findings were significant for the correlation between BMI and OSA. Although this crucial point was not covered in our study, it had been addressed in previous research, which might have been a limitation and something that could have been explored further.

Moreover, Logan et al. (17) reported a case series of 41 patients with resistant hypertension where 83% of patients with resistant hypertension had OSA. This study could not exclude other associated risk factors with resistant hypertension as this study lacked a control group with AHI >10 events/hour (17). Similarly, Pedrosa et al. reported that 64 % of resistant hypertension patients had OSA with AHI >15 events/hours (42). However, the prevalence of resistant hypertension in OSA patients is less studied. Our study demonstrates that the prevalence of resistant hypertension in OSA is 4.1 times more than in non-OSA patients, which was relatively small compared with previous studies (17, 42). Ruttanaumpawan et al. (32) demonstrate relatable findings with our study. OSAS patients have 3.99 times higher odds of developing resistant hypertension than non-OSAS, including other confounding factors, such as age, gender, and obesity.

Also, the clinical observation suggests OSAS patients have higher sympathetic nerve activity as measured by 24 h of urinary catecholamine excretion (43). The relevant survey reported that 70–83% of OSA patients suffered from resistant hypertension (17). Similarly, untreated OSAS patients have reduced efficacy of anti-hypertensive drugs (2, 17).

The patient's gender and OSA are considered significant factors for resistant hypertension. Goncalves et al. (19) had only reported OSAS risk for resistant hypertension across males and females. Resistant hypertension occurred most in males (OR: 5.5 [1.26, 23.9], P:0.023) than in females (OR: 4.2 [1.6, 10.59, p:0.002]) with OSAS. In the population-based case–control study, Hedner et al. (44) reported a similar finding that males have a higher risk for resistant hypertension than females.

Heterogeneity occurred from the diverse statistical methodology of clinical study. Thus, heterogeneity analysis is significant for validation of outcome of meta-analysis. The only outcome from multivariate analysis in Cai et al. (31) with other demonstrated substantial heterogeneity. Thus, our study does not have any heterogeneity level after omitting the outcome from Cai et al. (31). Thus, our meta-analysis demonstrated a robust outcome with no heterogeneity as shown in Figures 4, 6. Potential confounding factors such as age and obesity are significantly associated with hypertension and OSAS. We performed the analysis with (Figure 6) and without (Figure 4) consideration of these confounding factors. Both analyses demonstrated a significant association between resistant hypertension and OSA with no heterogeneity. In addition to overall effects, all the eligible studies favored the risk of resistant hypertension from OSAS.

### Limitations

This systematic review and meta-analysis have several limitations. Most of the eligible studies are observational studies that may restrict the persuasive value of our conclusion. In addition, there may be a chance of selection or reporting bias in an observational study. Only seven studies were eligible for analysis, and the number of patients/studies was small. Also, some hidden confounding factors such as different blood pressure instruments and polysomnography were never assessed. The subgroup analysis could not be performed as most studies failed to report primary data between the association of OSA and resistant hypertension based on age, gender, body mass index, and other comorbidities. To shed light on another potential limitation of our study would be to highlight that we did not consider the BMI and weight changes to cause OSA in patients, something which has been worked upon in previous literature and has been emphasized to play a crucial in causing OSA and hypertension. Hence, it could be listed as a limitation as well.

## Conclusion

This study provides strong qualitative and quantitative evidence to demonstrate that patients with OSAS are at increased risk for resistant hypertension. This review demonstrated the consistency with outcomes across country-specific studies and the strength of the qualitative studies involving OSA in hypertension morbidity.

## Data availability statement

The original contributions presented in the study are included in the article/supplementary material, further inquiries can be directed to the corresponding author.

## Author contributions

Conceptualization: AA and YX. Methodology and investigation: AA, SN, and YX. Writing—original draft preparation: AA. Writing—review and editing: AA and SN. Visualization and supervision: YX. All authors reviewed the manuscript, gave their final approval, and agreed to be accountable for all aspects of work ensuring integrity and accuracy.

## References

[1] Qaseem A Holty JEC Owens DK Dallas P Starkey M Shekelle P. Management of obstructive sleep apnea in adults: a clinical practice guideline from the American college of physicians. Ann Intern Med. (2013) 159:471–83. 10.7326/0003-4819-159-7-201310010-00704 24061345

[2] Chobanian AV Bakris GL Black HR Cushman WC Green LA Izzo JL Jr . Seventh report of the joint national committee on prevention, detection, evaluation, and treatment of high blood pressure. Hypertension. (2003) 42:1206–52. 10.1161/01.HYP.0000107251.49515.c2 14656957

[3] O'Connor GT Caffo B Newman AB Quan SF Rapoport DM Redline S . Prospective study of sleep-disordered breathing and hypertension: the sleep heart health study. Am J Respir Crit Care Med. (2009) 179:1159–64. 10.1164/rccm.200712-1809OC 19264976 PMC2695498

[4] Peppard PE Young T Barnet JH Palta M Hagen EW Hla KM. Increased prevalence of sleep-disordered breathing in adults. Am J Epidemiol. (2013) 177:1006–14. 10.1093/aje/kws342 23589584 PMC3639722

[5] Lin CM Davidson TM Ancoli-Israel S. Gender differences in obstructive sleep apnea and treatment implications. Sleep Med Rev. (2008) 12:481–96. 10.1016/j.smrv.2007.11.003 18951050 PMC2642982

[6] Haas DC Foster GL Nieto FJ Redline S Resnick HE Robbins JA . Age-dependent associations between sleep-disordered breathing and hypertension: importance of discriminating between systolic/diastolic hypertension and isolated systolic hypertension in the Sleep Heart Health Study. Circulation. (2005) 111:614–21. 10.1161/01.CIR.0000154540.62381.CF 15699282

[7] Walia HK Li H Rueschman M Bhatt DL Patel SR Quan SF . Association of severe obstructive sleep apnea and elevated blood pressure despite antihypertensive medication use. J Clin Sleep Med. (2014) 10:835–43. 10.5664/jcsm.3946 25126027 PMC4106935

[8] Pasha K Towhiduzzaman M Manwar A Jahan MU. Resistant hypertension—An update. Mymensingh Med J. (2015) 24:434–43. 26007281

[9] Mokhlesi B Hagen EW Finn LA Hla KM Carter JR Peppard PE. Obstructive sleep apnoea during REM sleep and incident non-dipping of nocturnal blood pressure: a longitudinal analysis of the Wisconsin sleep cohort. Thorax. (2015) 70:1062. 10.1136/thoraxjnl-2015-207231 26307037 PMC7888359

[10] Carey RM Calhoun DA Bakris GL Brook RD Daugherty SL Dennison-Himmelfarb CR . Resistant hypertension: detection, evaluation, and management: a scientific statement from the American heart association. Hypertension. (2018) 72:e53–90. 10.1161/HYP.0000000000000084 30354828 PMC6530990

[11] Acelajado MC Hughes ZH Oparil S Calhoun DA. Treatment of resistant and refractory hypertension. Circ Res. (2019) 124:1061–70. 10.1161/CIRCRESAHA.118.312156 30920924 PMC6469348

[12] Persell SD. Prevalence of resistant hypertension in the United States, 2003–2008. Hypertension. (2011) 57:1076–80. 10.1161/HYPERTENSIONAHA.111.170308 21502568

[13] Egan BM Zhao Y Axon RN Brzezinski WA Ferdinand KC. Uncontrolled and apparent treatment resistant hypertension in the United States, 1988 to 2008. Circulation. (2011) 124:1046–58. 10.1161/CIRCULATIONAHA.111.030189 21824920 PMC3210066

[14] Carey RM Sakhuja S Calhoun DA Whelton PK Muntner P. Prevalence of apparent treatment-resistant hypertension in the United States. Hypertension. (2019) 73:424–31. 10.1161/HYPERTENSIONAHA.118.12191 30580690 PMC6693520

[15] Naseem R Adam AM Khan F Dossal A Khan I Khan A . Prevalence and characteristics of resistant hypertensive patients in an Asian population. Indian Heart J. (2017) 69:442–6. 10.1016/j.ihj.2017.01.012 28822508 PMC5560875

[16] Muxfeldt ES Margallo VS Guimarães GM Salles GF. Prevalence and associated factors of obstructive sleep apnea in patients with resistant hypertension. Am J Hypertens. (2014) 27:1069–78. 10.1093/ajh/hpu023 24705438

[17] Logan AG Perlikowski SM Mente A Tisler A Tkacova R Niroumand M . High prevalence of unrecognized sleep apnoea in drug-resistant hypertension. J Hypertens. (2001) 19:2271–7. 10.1097/00004872-200112000-00022 11725173

[18] Calhoun DA Nishizaka MK Zaman MA Harding SM. Aldosterone excretion among subjects with resistant hypertension and symptoms of sleep apnea. Chest. (2004) 125:112–7. 10.1378/chest.125.1.112 14718429

[19] Gonçalves SC Martinez D Gus M de Abreu-Silva EO Bertoluci C Dutra I . Obstructive sleep apnea and resistant hypertension: a case-control study. Chest. (2007) 132:1858–62. 10.1378/chest.07-1170 18079220

[20] Lavie P Herer P Hoffstein V. Obstructive sleep apnoea syndrome as a risk factor for hypertension: population study. BMJ. (2000) 320:479–82. 10.1136/bmj.320.7233.479 10678860 PMC27290

[21] Muxfeldt ES Margallo V Costa LM Guimarães G Cavalcante AH Azevedo JC . Effects of continuous positive airway pressure treatment on clinic and ambulatory blood pressures in patients with obstructive sleep apnea and resistant hypertension: a randomized controlled trial. Hypertension. (2015) 65:736–42. 10.1161/HYPERTENSIONAHA.114.04852 25601933

[22] Thunström E Manhem K Rosengren A Peker Y. Blood pressure response to losartan and continuous positive airway pressure in hypertension and obstructive sleep apnea. Am J Respir Crit Care Med. (2016) 193:310–20. 10.1164/rccm.201505-0998OC 26414380

[23] Dempsey JA Veasey SC Morgan BJ O'Donnell CP. Pathophysiology of sleep apnea. Physiol Rev. (2010) 90:47–112. 10.1152/physrev.00043.2008 20086074 PMC3970937

[24] Nieto FJ Young TB Lind BK Shahar E Samet JM Redline S . Association of sleep-disordered breathing, sleep apnea, and hypertension in a large community-based study. JAMA. (2000) 283:1829–36. 10.1001/jama.283.14.1829 10770144

[25] Appleton SL Vakulin A Martin SA Lang CJ Wittert GA Taylor AW . Hypertension is associated with undiagnosed OSA during rapid eye movement sleep. Chest. (2016) 150:495–505. 10.1016/j.chest.2016.03.010 27001264

[26] Shamseer L Moher D Clarke M Ghersi D Liberati A Petticrew M . Preferred reporting items for systematic review and meta-analysis protocols (PRISMA-P) 2015: elaboration and explanation. Bmj. (2015) 349:7647. 10.1136/bmj.g7647 25555855

[27] Borenstein M Hedges L Higgins J Rothstein H. Comprehensive Meta-Analysis 3ed. Englewood: Biostat (2013).

[28] The EndNote Team. EndNote 20 ed. Philadelphia, PA: Clarivate (2021).

[29] Moher D Liberati A Tetzlaff J Altman DG. Preferred reporting items for systematic reviews and meta-analyses: the PRISMA statement. Int J Surg. (2010) 8:336–41. 10.1016/j.ijsu.2010.02.007 20171303

[30] Abdel-Kader K Dohar S Shah N Jhamb M Reis SE Strollo P . Resistant hypertension and obstructive sleep apnea in the setting of kidney disease. J Hypertens. (2012) 30:960–6. 10.1097/HJH.0b013e328351d08a 22388231 PMC3771863

[31] Cai A Zhang J Wang R Chen J Huang B Zhou Y . Joint effects of obstructive sleep apnea and resistant hypertension on chronic heart failure: a cross-sectional study. Int J Cardiol. (2018) 257:125–30. 10.1016/j.ijcard.2017.10.089 29506683

[32] Ruttanaumpawan P Nopmaneejumruslers C Logan AG Lazarescu A Qian I Bradley TD. Association between refractory hypertension and obstructive sleep apnea. J Hypertens. (2009) 27:1439–45. 10.1097/HJH.0b013e32832af679 19421073

[33] Wu Y Hu G Pan F Liu J Mo X Xie Y . Obstructive sleep apnea hypopnea syndrome was a risk factor for uncontrolled hypertension in adult snorers in South China. Clin Exp Hypertens. (2016) 38:429–34. 10.3109/10641963.2016.1151525 27359186

[34] Drager LF Genta PR Pedrosa RP Nerbass FB Gonzaga CC Krieger EM . Characteristics and predictors of obstructive sleep apnea in patients with systemic hypertension. Am J Cardiol. (2010) 105:1135–9. 10.1016/j.amjcard.2009.12.017 20381666

[35] Egger M Smith GD Schneider M Minder C. Bias in meta-analysis detected by a simple, graphical test. Bmj. (1997) 315(7109):629-34. 10.1136/bmj.315.7109.629 9310563 PMC2127453

[36] Lavie L. Obstructive sleep apnoea syndrome—An oxidative stress disorder. Sleep Med Rev. (2003) 7:35–51. 10.1053/smrv.2002.0261 12586529

[37] Pratt-Ubunama MN Nishizaka MK Boedefeld RL Cofield SS Harding SM Calhoun DA. Plasma aldosterone is related to severity of obstructive sleep apnea in subjects with resistant hypertension. Chest. (2007) 131:453–9. 10.1378/chest.06-1442 17296647

[38] Pimenta E Stowasser M Gordon RD Harding SM Batlouni M Zhang B . Increased dietary sodium is related to severity of obstructive sleep apnea in patients with resistant hypertension and hyperaldosteronism. Chest. (2013) 143:978–83. 10.1378/chest.12-0802 23288434 PMC3616687

[39] Hou H Zhao Y Yu W Dong H Xue X Ding J . Association of obstructive sleep apnea with hypertension: a systematic review and meta-analysis. J Global Health. (2018) 8:405. 10.7189/jogh.08.010405 29497502 PMC5825975

[40] Carratù P Di Ciaula A Dragonieri S Ranieri T Matteo Ciccone M Portincasa P . Relationships between obstructive sleep apnea syndrome and cardiovascular risk in a naïve population of southern Italy. Int J Clin Pract. (2021) 75:e14952. 10.1111/ijcp.14952 34610197 PMC9285080

[41] Carratù P Ventura VA Maniscalco M Dragonieri S Berardi S Ria R . Echocardiographic findings and plasma endothelin-1 levels in obese patients with and without obstructive sleep apnea. Sleep Breath. (2016) 20:613–9. 10.1007/s11325-015-1260-5 26385777

[42] Pedrosa RP Drager LF Gonzaga CC Sousa MG de Paula LK Amaro AC . Obstructive sleep apnea: the most common secondary cause of hypertension associated with resistant hypertension. Hypertension. (2011) 58:811–7. 10.1161/HYPERTENSIONAHA.111.179788 21968750

[43] Luik AI Direk N Zuurbier LA Hofman A Van Someren EJW Tiemeier H. Sleep and 24-h activity rhythms in relation to cortisol change after a very low-dose of dexamethasone. Psychoneuroendocrinology. (2015) 53:207–16. 10.1016/j.psyneuen.2015.01.011 25635613

[44] Hedner J Bengtsson-Boström K Peker Y Grote L Råstam L Lindblad U. Hypertension prevalence in obstructive sleep apnoea and sex: a population-based case–control study. Eur Resp J. (2006) 27:564. 10.1183/09031936.06.00042105 16507857

